# The Allocation Method for Personal Protective Equipment in the Emerging Infectious Disease Environment

**DOI:** 10.3389/fpubh.2022.904569

**Published:** 2022-05-27

**Authors:** Sha-lei Zhan, Xinyi Gu, Yong Ye, Yen-Ching Chuang

**Affiliations:** ^1^School of Management and E-Business, Zhejiang Gongshang University, Hangzhou, China; ^2^Institute of Public Health & Emergency Management, Taizhou University, Taizhou, China; ^3^Business College, Taizhou University, Taizhou, China

**Keywords:** humanitarian logistics, emerging infectious diseases, personal protective equipment, multi-objective optimization, branch and bound method

## Abstract

The COVID-19 pandemic gives humankind a lesson that the outbreak of an emerging infectious disease (EID) is sudden and uncertain. Accurately mastering its dynamics and putting forward an efficient and fair humanitarian logistics plan for personal protective equipment (PPE) remains difficult. This study examines the decision making for humanitarian logistics to answer the question that how to coordinate fairness and efficiency when facing supply-demand imbalance during humanitarian logistics planning in an EID environment. The main contributions include two aspects: (1) The victims' losses in terms of fairness and efficiency in receiving PPE are jointly explored by evaluating their bearing capacity evolution, and then a novel loss function is built to search for a reasonable compromise between fairness and efficiency. (2) A multi-objective optimization model is built, which is solved using the combined use of goal programming approach and improved branch and bound method. Finally, the practicability of the proposed model is tested by an EID case study. The potential advantages of the proposed model and improved approach are discussed.

## Introduction

In the past decade, public health emergencies have caused widespread concern around the world, and emerging infectious diseases (EIDs) have become intractable in terms of prevention and control of public health emergencies in all countries (1). The COVID-19 pandemic, broke out worldwide in 2020 and lasted 2 years, is the most prominent example which has led to the numerous infected cases worldwide (2). Evidence from different cases shows that it can spread through close contact with infected persons and contaminated surfaces (3). It is important to note that the personal protective equipment (PPE) is the last barrier to prevent people being infected (4). Therefore, the appropriate humanitarian logistics planning for PPE is of great significance, such as mitigating the potential disease spread risk, weakening the impacts on victims' losses resulting from an undergoing pandemic, and therefore benefiting the public safety and social welfare.

As one of the most impactive public health emergencies, an EID refers to such an infectious disease that has a sudden occurrence in a short time (like a “black swan event”), spreads across a large region and results in a large number of cases (5). It is very likely to cause catastrophic damages to human health and even deprive their life. There are following three characteristics of an EID. First, once an EID breaks out, it may spread rapidly and widely, so that it not only brings the physical damage to the infected people, but also does harm to those who are uninfected yet but psychologically distressed by the potential risk (6). Therefore, when facing a humanitarian logistics planning problem for PPE, the traditional cost function such as unmet-demand cost cannot represent the whole loss, and a more proper loss function is worth exploring. Second, the dynamics and mutability of EID evolution reduce the accuracy and reliability of gathered data and information (7). Under this premise, exploring the law of EID evolution and quantification of the losses caused by EID are both not easy. More particularly, the quantification of losses caused by EID immediately influences the follow-up PPE allocation decision-making quality. Third, there is an imbalance between PPE demand and supply under an EID scenario (8, 9). PPE is usually insufficient, therefore such an imbalance is often manifested by supply shortage during EID development, which will cause the tradeoffs between the fairness and efficiency issues when designing humanitarian logistics planning for PPE. Moreover, the quantification of losses caused by EID is also closely-related to PPE supply-demand imbalance.

By jointly considering the all three characteristics, two core questions then arise as follows.

1. How Should the Losses Caused by EID be Exactly Quantified by a Proper Approach in a More Scientific way?
2. How Should the Quantification of Losses Caused by EID be Integrated With the Tradeoffs Between the Fairness and Efficiency Issues?

This study seeks to answer these two questions by exploring the victims' losses in terms of fairness and efficiency in receiving PPE and building a novel loss function to evaluate their bearing capacity evolution. In previous studies, fairness and efficiency are considered respectively in an independent way, e.g., represented by two conflicting objectives (10–12). Then, search for a proper compromise between them is difficult due to the non-comparability between any two non-dominated solutions in a Pareto Front. However, our proposed bearing capacity evolution can overcome this drawback and reach a real compromise between fairness and efficiency in humanitarian logistics.

Regarding the modeling and solution, a multi-objective optimization model is built, which is solved by the combined use of the goal programming approach and the improved branch and bound (B&B) method. Although the goal programming approach is frequently employed in the previous studies involving multi-objective optimization issues, how to design the following-up solution procedure to simultaneously pursue efficiency and accuracy is worth exploring, especially when facing a large-scale problem (11, 13). Moreover, integrating the goal programming approach and the improved B&B method is also rare in the previous humanitarian-logistics-related studies.

The remainder of this study is organized as follows. Section 2 conducts a literature review about the decision making on humanitarian logistics for PPE. Section 3 introduces the network structure, the mathematical model, and the solution procedure for humanitarian logistics for PPE. Section 4 presents the numerical results and Section 5 discusses the advantages of the proposed model and solution. Section 6 presents the conclusions and future directions.

## Literature Review

Many related studies have been conducted and made different contributions. The main research status is introduced as follows.

### Humanitarian Logistics

Humanitarian logistics has become a popular topic in the last two decades, comprehensive reviews about humanitarian logistics have been proposed by Altay and Green (14), Caunhye et al. (15) and Besiou and Van Wassenhove (1). Early research focuses on vehicle routing problems (16, 17) and facility location problems (18). Then, an increasing number of research turns the eyes to the integration of those subproblems, for instance, Zhan et al. (19), Rodríguez-Espíndola et al. (20) and Seraji et al. (21) both discuss the location-allocation problem, while Moreno et al. (22) studies the location-routing-allocation problem related to humanitarian logistics. Duhamel et al. (23) and Shavarani (24) both deal with the location-routing problem to optimize the humanitarian relief distribution decision-making. Eisenhandler and Tzur (11) address the routing-allocation problems to guide the decision-making in collecting food donations from suppliers in the food industry and delivering them to humanitarian relief agencies that serve individuals in need. Most of the aforementioned studies do not consider dynamics such as emergencies evolution and information updates. Not-enough-accurate and even incorrect information may cause catastrophic consequences. Therefore, considering the dynamics in humanitarian logistics has important practical significance. Early studies pay considerable attention to forecasting the evolution law of emergencies, but ignore the follow-up decision making on humanitarian logistics. For example, Sheu (25) focuses on the relief demand management based on an imperfect information environment, but omits relief allocation decision-making. To make up this lack, in another study of Sheu (26), he deduces the “perception-attitude-resilience” evolution relationship of demanders through psychological and cognitive theories to aid the relief goods allocation. Lu et al. (27) present a rolling horizon approach enabling the established model to conform to the evolution law of demand information. Haghi et al. (28) consider the changes in demand to build a model for the distribution of relief supplies and transfer of the wounded. Recent studies combine the dynamics and the follow-up decision-making together to present an integrated event-oriented relief logistics plan, for instance, Zhang et al. (29) examine the emergency resource allocation problem by simultaneously considering three stages including pre-, primary- and secondary-event stage. Cao et al. (30) address a dynamic multi-time-period relief distribution model considering supplies uncertainty, hierarchal decision levels and conflicting objectives. Uichanco (31) develops an integrated stochastic prepositioning model in which the probabilities of demand and supply damage are both dependent on the event outcome.

The distribution of humanitarian supplies in response to EID is also very important, but the research on this issue is relatively limited. Most research still focuses on the humanitarian logistics of large-scale natural disasters without considering the characteristics of EID (33–35). Only a few studies have considered the dynamics and mutability of EID evolution. Zaric and Brandeau (36) develop a dynamic resource allocation model for epidemic control over multiple time periods to interventions that affect multiple populations. Wang et al. (37) construct a multi-objective stochastic programming model with time-varying demand based on the epidemic diffusion rule, and Genetic algorithm and Monte Carlo simulation are used to solve the problem. He and Liu (38) present a new medical emergency logistics model based on the time-varying forecasting and relief distribution to deal with public health emergencies. Büyüktahtakin et al. (39) build an epidemics-logistics mixed-integer programming model to determine the optimal amount, timing and location of resources to control an infectious disease outbreak. Qin et al. (40) propose an even swaps method based on the prospect theory with hesitant fuzzy linguistic term sets to put forward emergency logistics plans under the COVID-19 pandemic outbreak. However, the dynamics of demand depicted in these studies are related to time or scenario, not to the victims' bearing capacity which is more direct and accurate. Therefore, the present study aims to overcome this drawback and reexamine the dynamics of demand by building the victims' losses function based on their bearing capacity evolution.

### Multi-Objective Optimization

Numerous studies formulate humanitarian logistics problem as a multi-objective optimization model. Traditional studies have optimized over efficiency-related and fairness-related criteria. For example, Tzeng et al. (41) propose the time, cost and demand satisfaction as main pursued objectives, Vitoriano et al. (42) consider more highly-relevant criteria, such as reliability, security, priority and ransack probability, Huang et al. (43) add a new metric called efficacy which denotes the extent to which the goals of quick and efficient logistics are met, Tofighi et al. (44) focus on the timely and fair provision of aid and propose a multi-objective model from two novel aspects including egalitarian aspect and utilitarian aspect. In comparison, the recent studies propose multiple criteria from much wider viewpoints. Roughly speaking, the utility (45), risk (46), sustainability-related metrics such as carbon-emissions (30) are main new-added criteria. In some recent research, the criteria are deepened in detail to the operational level. For instance, Zhou et al. (47) tackle two objectives involving minimizing the unmet demand and minimizing the risk of choosing the damaged road for dynamic emergency resource scheduling problems, Cao et al. (13) pursue simultaneously the maximization of the victims' satisfaction and minimization of the deviation on satisfaction in humanitarian relief distribution, Uichanco (31) coordinates two objectives involving minimizing the unmet demand and minimizing the unmet proportion of demand for prepositioning humanitarian relief items, Mohammadi et al. (32) consider three objectives where the first objective minimizes the total logistics costs and the third one minimizes the variation between upper and lower bounds of transportation cost. According to incomplete statistical survey involving the recent three-year studies on humanitarian relief logistics optimization, the timeliness (e.g. minimizing total time) and fairness (e.g. minimizing unmet demand) are still the main concerns, but how to coordinate them and search for a proper compromise is still under discussion.

Regarding the methodologies for multi-objective optimization, the Pareto front is worth exploring by some popular methods, such as ε-constrained method (31, 48), augmented ε-constrained method (10) and fuzzy goal constraint approach (29). When facing large-scale numerical examples, the solution approaches, developing two main streams which include either exact approach and the heuristics, have been widely examined. With respect to exact approach, Dalal and Üster (49) build a robust optimization model to aid emergency relief supply planning and solve the model by Benders decomposition method, Cao et al. (30) employ a hybrid global criterion method by incorporating a primal-dual algorithm, expected value and branch-and-bound approach to solve the multi-period humanitarian relief distribution model, Mohammadi et al. (32) solve the humanitarian facility location and vehicle routing model by using GAMS software and the BARON optimization solver. Regarding heuristics, the evolutionary algorithm (47), genetic algorithm (13, 50), bi-level algorithm (51), colony optimization algorithm (52) and NSGA-II (12) are employed in different multi-objective optimization studies. The common limits of the above studies lie in the non-comparability between any two non-dominated solutions in a Pareto Front. However, our proposed bearing capacity evolution, and the follow-up solution approach integrating the goal programming approach and the improved B&B method can overcome this drawback and reach a real compromise between fairness and efficiency in humanitarian logistics.

## Humanitarian Logistics Model Based on Victims' Bearing Capacity Evolution

### Problem Description

**Figure 1:**
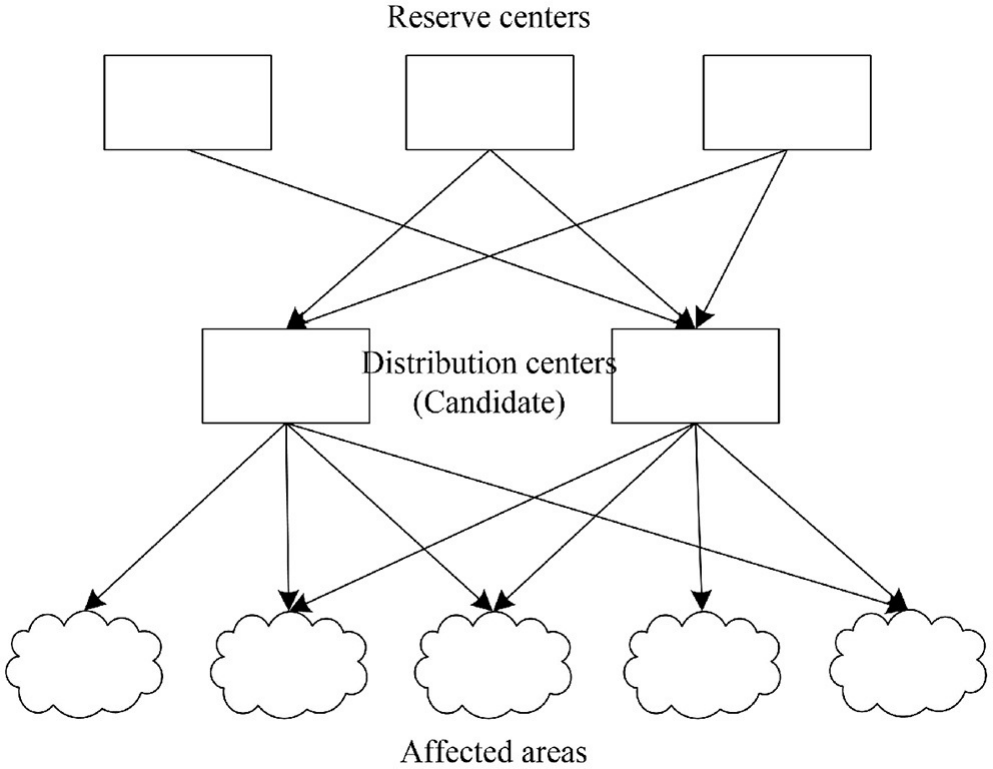
The humanitarian logistics network.

This study examines a three-layer location-allocation network considering multiple PPEs and multiple vehicles. Three network layers, including reserve centers, distribution centers (DCs), and affected areas, respectively have different roles, capacities, and locations. Reserve centers denote warehouses, with fixed, predetermined locations and capacities, that store multiple PPEs and emergency vehicles. Multiple PPEs shows actual demand in an EID scenario, such as masks, gloves, gown and face shields, which have different importance to victims. Multiple vehicles refer to the diversified transportation modes that combine several kinds of vehicles with different loading weights. DCs gather and integrate the multiple PPEs and multiple vehicles transported from the reserve centers, and then deliver them to the affected areas. Borrowing the idea of Li et al. (53), the locations of DCs are undetermined, and needed to be chosen from several candidate locations which have different capacities for PPEs and vehicles. Decision makers have to select optimal locations for DCs before an EID occurs and allocate PPEs after the EID. Multiple objectives should be addressed during decision making. Figure 1 shows the network structure.

### The Model

#### Sets and Parameters

*H* is set of reserve centers, *h* ∈ *H*.
*I* is set of candidate locations of DCs, *i* ∈ *I*.
*J* is set of affected areas, *j* ∈ *J*.
*K* is set of PPEs, *k* ∈ *K*.
*M* is set of vehicle types, *m* ∈ *M*.
*N* is set of objective functions, *n* ∈ *N*.
*D*_*hij*_ is the distance from reserve center *h* to DC *i* and then to affected area *j* (m).
*C* is the maximum coverage range of vehicles in the reserve centers, that is, the longest transportation distance that vehicles can travel (m).
*w*_*k*_ is the weight, reflecting the importance of PPE *k* to victims.
*V*_*m*_ is the maximum loading weight of vehicle *m* (kg/vehicle).
*U*_*hm*_ is the supply of vehicle *m* in the reserve center *h* (vehicles).
*W*_*hk*_ is the supply of PPE *k* in the reserve center *h* (kg).
*P*_*im*_ is the maximum capacity of vehicle *m* in DC *i* (vehicles).
*Q*_*ik*_ is the maximum capacity of PPE *k* in DC *i* (kg).
*d*_*jk*_ is the demand quantity of the affected area *j* for PPE *k* (kg).
*f*_*m*_ is the transportation cost of vehicle *m* per unit distance (yuan/m).
*e*_*m*_ is the transportation time of vehicle *m* per unit distance (s/m).
*c*_*i*_ is the renting cost of DC *i* (yuan).

#### Decision Variables

- *z*_*i*_ is location variable, indicates whether the candidate location *i* is used as the DC.
- *x*_*hijm*_ is routing variable, indicates the number of vehicle *m* to transport PPE from the reserve center *h* to the DC *i* and then to the affected area *j* (vehicles).
- *y*_*hijkm*_ is allocation variable, indicates the quantity of PPE *k* which are delivered by vehicles *m* from the reserve center *h* to the DC *i* and then to the affected area *j* (kg).

#### Objective Function **G**_**1**_: Minimizing Total Losses in Waiting for PPE

**Figure 2:**
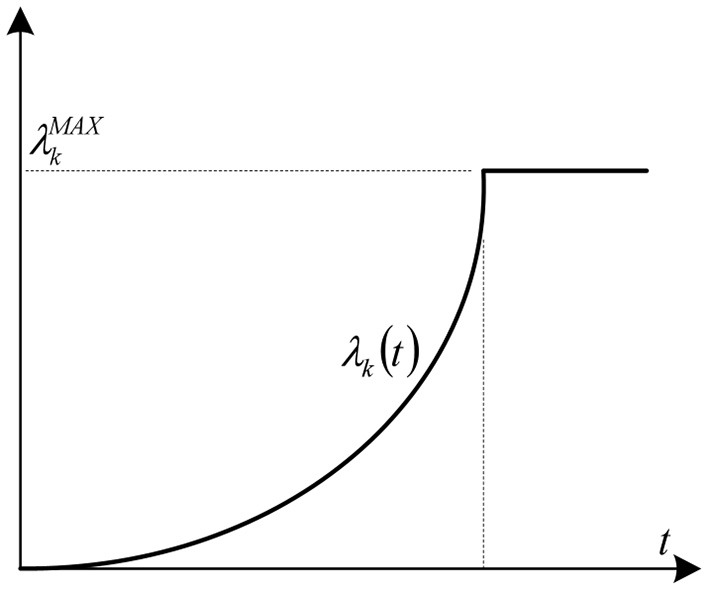
Unit cost curve of victims' waiting for PPE *k*.

In an EID scenario, victims' bearing capacity evolution is actually a process of loss accumulation. Long waiting time leads to increase in victims' losses. In this study, such losses are quantified by costs. Suppose the cost curve of victims waiting for one unit of PPE *k* is λ_*k*_(*t*), where *t* is the waiting time. When facing PPE supply shortage, victims' physiological and psychological bearing capacity continuously decreases as time passes. This decline rate is continuously intensifying. Hence, the cost curve increases in a limited time range by an increasing growth rate (see Figure 2). In other words, $\lambda_{k}^{{}^{\prime }}\left(t\right)>0$ and $\lambda_{k}^{{}^{\prime \prime }}\left(t\right)>0$. Victims that do not receive any PPE for a long time will receive the maximum cost after a certain time point. At this point onward, correspondingly, the cost curve of victims becomes a horizontal straight line ($\lambda_{k}^{MAX}$).

A loss function (*L*_*jk*_) that uses waiting time as the independent variable is designed as follows, which reflects victims' total losses caused by waiting for PPE *k* in the affected area *j*.


(1) $$\begin{array}{l} L_{jk}=\left(d_{jk}-\underset{h}{\sum }\underset{i}{\sum }\underset{m}{\sum }y_{hijkm}\right)\cdot \lambda_{k}^{MAX}\\ \;+\underset{h}{\sum }\underset{i}{\sum }\underset{m}{\sum }y_{hijkm}\cdot \lambda_{k}\left(t\right) \end{array}$$

where


(2) $$\begin{array}{l} t=e_{m}\cdot D_{hij}. \end{array}$$

This loss function contains two parts. The first part indicates victims' losses caused by unmet demands (influences the realization of the fairness objective), and can be viewed as victims' losses caused by infinite delay of PPE. The second part denotes victims' losses caused by delayed supply (influences the realization of the efficiency objective), and can also be viewed as victim's losses caused by finite delay of PPE. Therefore, this loss function measures both the fairness and efficiency, and is in fact, a compromise between these two conflicting objectives.

After building the loss function, the objective function *G*_1_ can be formulated as follows.


(3) $$\begin{array}{l} G_{1}=min\underset{j}{\sum }\underset{k}{\sum }L_{jk}\cdot w_{k}. \end{array}$$

#### Objective Function **G**_**2**_: Minimizing Total Logistics Costs


(4) $$\begin{array}{l} G_{2}=min\left(\underset{i}{\sum }c_{i}\cdot z_{i}+\underset{h}{\sum }\underset{i}{\sum }\underset{j}{\sum }\underset{m}{\sum }D_{hij}\cdot f_{m}\cdot x_{hijm}\right). \end{array}$$

This objective function also contains two parts. The first part is the renting cost of DCs, whereas the second is the transportation costs of PPEs.

#### Constraints


(5) $$\begin{array}{l} x_{hijm}=\{\begin{array}{l} \;\\ \geq 0,D_{hij}\leq C\\ \;\\ =0,D_{hij}>C\\ \; \end{array}\;,\forall h,i,j,m. \end{array}$$

(6) $$\begin{array}{l} \underset{i}{\sum }\underset{j}{\sum }x_{hijm}\leq U_{hm},\forall h,m. \end{array}$$

(7) $$\begin{array}{l} \underset{i}{\sum }\underset{j}{\sum }\underset{m}{\sum }y_{hijkm}\leq W_{hk},\forall h,k. \end{array}$$

(8) $$\begin{array}{l} \underset{h}{\sum }\underset{j}{\sum }x_{hijm}\leq P_{im}\cdot z_{i},\forall i,m. \end{array}$$

(9) $$\begin{array}{l} \underset{h}{\sum }\underset{j}{\sum }\underset{m}{\sum }y_{hijkm}\leq Q_{ik}\cdot z_{i},\forall i,k. \end{array}$$

(10) $$\begin{array}{l} \underset{k}{\sum }y_{hijkm}\leq V_{m}\cdot x_{hijm},\forall h,i,j,m. \end{array}$$

(11) $$\begin{array}{l} \underset{h}{\sum }\underset{i}{\sum }\underset{m}{\sum }y_{hijkm}\leq d_{jk},\forall j,k. \end{array}$$

(12) $$\begin{array}{l} z_{i}=0\;or\;1,\forall i. \end{array}$$

(13) $$\begin{array}{l} x_{hijm}\text{ is non}-\text{negative integer},\;\forall h,i,j,m. \end{array}$$

(14) $$\begin{array}{l} y_{hijkm}\geq 0,\forall h,i,j,k,m. \end{array}$$

Constraint (5) involves vehicle coverage, which indicates that each vehicle is only responsible for the delivery of PPEs to the affected area within a permitted distance. This constraint not only ensures the timeliness but also the quality and safety of PPEs. In an EID scenario, long-distance transportation is often unreliable because of the implementation of lockdown policies. Constraints (6) and (7) concern the reserve capacity of reserve centers that represent the maximum supply of vehicles and PPEs in reserve centers. Constraints (8) and (9) concern the maximum capacity of DCs for routing vehicles and allocating PPEs respectively. In addition, only chosen DCs are ensured to have flows of vehicles and PPEs. Constraint (10) is the maximum load capacity of vehicles for filling with PPEs. Constraint (11) indicates that the quantity of delivered PPEs is no more than the demand of victims, because excessive delivery is a considerable waste given the premise of shortage of PPEs. Finally, constraints (12)–(14) define the value ranges of decision variables.

### Solution Procedure

The proposed model is obviously a multi-objective optimization model and a complicated mixed-integer programming model if being applied to a large-scale numerical example. To simultaneously deal with these two within a reasonable runtime and with high accuracy is difficult in real-world practice. Under this consideration, this study puts forward a solution procedure by integrating goal programming approach and an improved B&B method.

In a humanitarian logistics optimization problem, the timely and effective delivery is vital. Cost minimization is often regarded as a less important goal. Therefore, two objective functions have clear priorities in terms of importance: *G*_1_ is far superior to *G*_2_. For such multi-objective programming problems, goal programming is an effective approach. Thus, the multi-objective programming model is transformed to a goal programming model, such as:


(15) $$\begin{array}{l} min\left(\lambda_{1}\cdot \theta_{1}^{+}+\lambda_{2}\cdot \theta_{2}^{+}\right) \end{array}$$

where λ_1_ and λ_2_ are priorities of the two objective functions. According to above analysis, λ_1_>>λ_2_. $\theta_{1}^{+}$ and $\theta_{2}^{+}$ are positive deviation variables, indicating that the original objective functions are larger than the quantity of ideal levels *R*_1_ and *R*_2_. Accordingly, negative deviation variables $\theta_{1}^{-}$ and $\theta_{2}^{-}$ exist, reflecting that the original objective functions are smaller than the quantity of ideal levels. However, given that the two objectives pursue minimization, only the positive deviation variables have to be minimized.

Apart from the constraints of the original model, other constraints include the following:


(16) $$\begin{array}{l} G_{n}+\theta_{n}^{-}-\theta_{n}^{+}=R_{n},\forall n \end{array}$$

(17) $$\begin{array}{l} \theta_{n}^{-}\cdot \theta_{n}^{+}=0,\forall n \end{array}$$

(18) $$\begin{array}{l} \theta_{n}^{-}\geq 0,\theta_{n}^{+}\geq 0,\forall n \end{array}$$

Equation (16) shows the relationship between the original objective function and the ideal level. Equation (17) is the expression of logical relations between the positive and negative deviation variables. When the objective function exceeds the ideal level, the positive deviation variables are larger than 0 and the negative deviation variables are certainly 0. By contrast, when the objective function does not exceed the ideal level, the negative deviation variables are larger than 0 and the positive deviation variables are certainly 0. If the objective function is only equal to the ideal level, then both the positive and negative deviation variables are 0. Equation (18) defines the value ranges of the positive and negative deviation variables.

After converted into goal programming, the original multi-objective optimization model becomes a large-scale mixed-integer programming model with a single objective. The B&B method is used as a solution by decomposing the mixed-integer programming model into a linear programming model and subsequently solving the linear programming model repeatedly. B&B, proposed by Land Doig and Dakin in the 1960s, is an algorithm design paradigm for discrete and combinatorial optimization problems, as well as mathematical optimization (54). However, a traditional B&B method cannot efficiently avoid exponential expansion of B&B tree when using tree search. This study uses the Xpress-MP software (developed by Dash Optimization), which uses an improved B&B method to avoid exponential node growth. A series of advanced algorithms used by Xpress-MP, such as pre-solving, cutting planes, branching variable selection, node preprocessing, and heuristics, can overcome the disadvantages of the traditional B&B method. Key procedures of improved B&B method are as follows. First, solve the LP relaxation, and save the basis of the optimal solution. Second, fix location variables to 0 if the corresponding routing and allocation variables are close to 0, and to 1 if they have relatively large values. Third, solve the resulting branched MIP problem. Fourth, if an integer feasible solution was found, save the value of the best solution. Five, restore the original problem by resetting all variables to their original bounds, and load the saved basis. Six, solve the original MIP problem, using the heuristic solution as cutoff value. Branches without roles in searching the best solution are cut; thus, the tree size will not expand quickly. This method has been proved suitable to solve large-scale complicated mixed-integer programming problems (55, 56).

## Example of Demonstration

### Example Description

A hypothetical EID occurs in the south region of Wenzhou, a southeastern city in China. Five areas are affected, namely, Rui'an, Pingyang, Cangnan, Wencheng, and Taishun. Demands for PPEs such as masks and protective clothing are very large (see Table 1), with weights 0.6 and 0.4, respectively. Officials gather vehicles (light, medium, and heavy vehicles) in reserve centers in Shiqu, Yongjia, and Yueqing to deliver PPEs to affected areas. Table 2 lists the quantity of PPEs and vehicles in reserve centers. Two candidate locations of DCs (DC1 and DC2) are present. Table 3 shows the capacities of these two DCs, and their renting costs are RMB 800,000 and 600,000, respectively. To ensure timeliness of PPE allocation, officials determine that the maximum transportation radius of vehicles is 150 km. Table 4 shows the other parameter settings. Additionally, the following is the cost function of waiting for PPEs:


$$\begin{array}{l} \lambda_{k}\left(t\right)=\{\begin{array}{c} \frac{t^{2}}{7315661},0\leq t\leq 604800\\ 50000,t>604800\\ \; \end{array}. \end{array}$$

**Table 1:** Demands in affected areas (1,000 kg).

**Affected areas**	**Masks**	**Protective clothing**
Rui'an	146	0
Pingyang	150	0
Cangnan	165	0.16
Wencheng	150	11.4
Taishun	320	3.04

**Table 2:** Supplies in reserve centers (1,000 kg; vehicles).

	**Masks**	**Protective**	**Light**	**Medium**	**Heavy**
		**clothing**	**vehicles**	**vehicles**	**vehicles**
Shiqu	500	6	30	15	10
Yongjia	150	2	10	5	2
Yueqing	300	4	20	10	5

**Table 3:** Capacities of DCs (1,000 kg; vehicles).

	**Masks**	**Protective**	**Light**	**Medium**	**Heavy**
		**clothing**	**vehicles**	**vehicles**	**vehicles**
DC1	800	10	50	25	12
DC2	600	8	40	20	10

**Table 4:** Other parameters.

	**Light**	**Medium**	**Heavy**
	**vehicles**	**vehicles**	**vehicles**
Load capacity of vehicles (kg/vehicle)	5,000	10,000	20,000
Unit transportation cost (yuan/m)	0.1	0.2	0.3
Unit transportation time (s/m)	0.06	0.07	0.09

### Optimal Plans for Humanitarian Logistics

**Figure 3:**
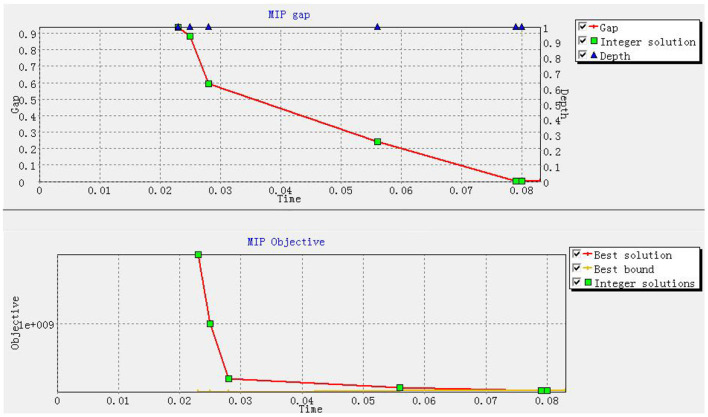
Gap between best solution and best bound.

The proposed model is solved by using the Xpress-MP 8.13 on a computer with 3.4 GHz CPU and 8 GB RAM. First, the single objective model of each objective function is solved independently, and the best values are used as the ideal levels of the two objectives. *R*_1_ = 1.26358*e*+8 and *R*_2_ = 0 are obtained and then both inserted into the goal programming model on the premise of λ_1_>>λ_2_, resulting in $\theta_{1}^{+}=0$ and $\theta_{2}^{+}=3.1975e+6$. This means that the minimum victims' losses caused by waiting for PPEs amount to RMB 0.126 billion, which fully reaches the ideal level. This also represents the compromise between fairness and timeliness during the humanitarian aid reaches an optimal level. Second, we regard the best values of two single-objective models as the lower bounds of two objectives, and the worst values [respectively resulting in 2.8222*e*+10 solved by $\max\underset{j}{\sum }\underset{k}{\sum }L_{jk}\cdot w_{k}$, and resulting in 3.7105*e*+6 solved by $\max\left(\underset{i}{\sum }c_{i}\cdot z_{i}+\underset{h}{\sum }\underset{i}{\sum }\underset{j}{\sum }\underset{m}{\sum }D_{hij}\cdot f_{m}\cdot x_{hijm}\right)]$ of two single-objective models as the upper bounds of two objectives. Then, using a specified formula such as (current value − lower bound)/(upper bound − lower bound), the percentage deviations of two objectives are respectively obtained as 0% and 86.17%. Although the second objective is not close to its ideal level, the absolute deviation (i.e. RMB 3.1975 million) is also acceptable when standing on the viewpoint of the humanitarians to pay the logistics costs of PPEs. Third, as shown in Figure 3, the total runtime is <1 s, which highly meets the demand of timeliness in humanitarian aid. Figure 3 also depicts the gap between the optimal solution and the optimal bound, that is, the optimal solution reaches the optimal bound, which infers the high accuracy of proposed solution method.

**Figure 4:**
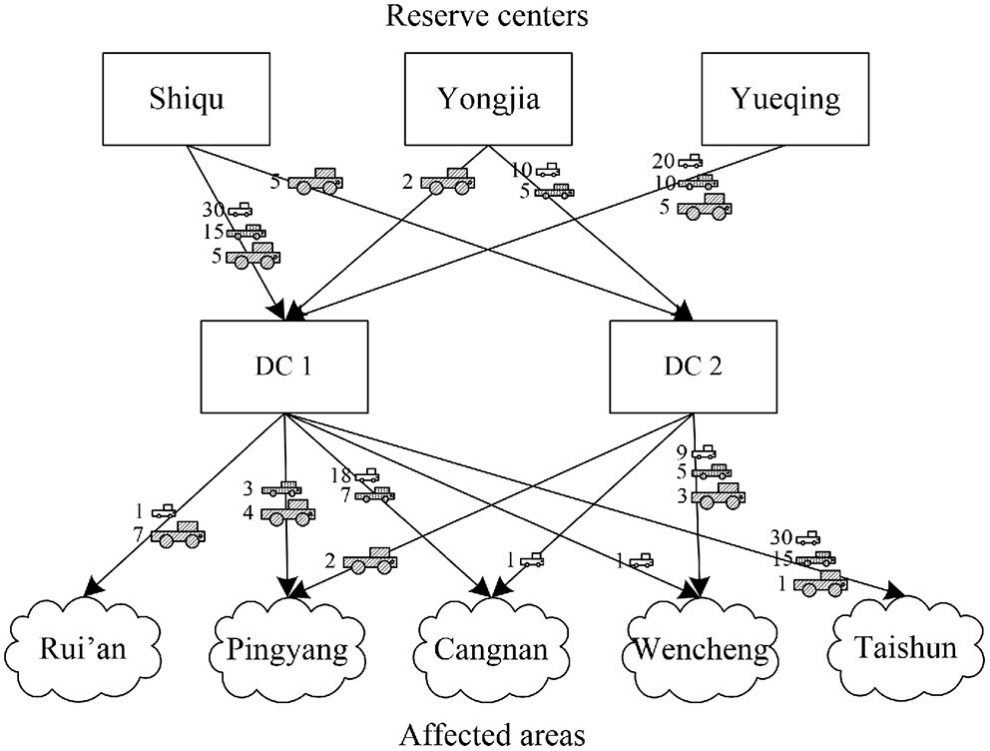
Optimal plans for humanitarian logistics.

Figure 4 shows specific plans for humanitarian logistics. Vehicles in all reserve centers have been used thoroughly. Both DC1 and DC2 are chosen to meet the demands of affected areas as much as possible. Note that Rui'an and Taishun only receive one vehicle route. According to further observation, Rui'an is slightly affected and does not need too many vehicles. Taishun is heavily affected and far away from DCs. Vehicles appointed to Taishun all come from Shiqu, because the maximum coverage of vehicles restricts long-term transportation. Therefore, two interesting insights are gleaned, as follows: (i) the best solution of PPE allocation is prioritizing heavily affected areas than slightly affected areas to realize the fairness consideration; (ii) the best solution ensures timeliness of PPE allocation through the maximum coverage range of vehicles.

## Discussion

### Tradeoffs Between the Two Objectives

**Figure 5:**
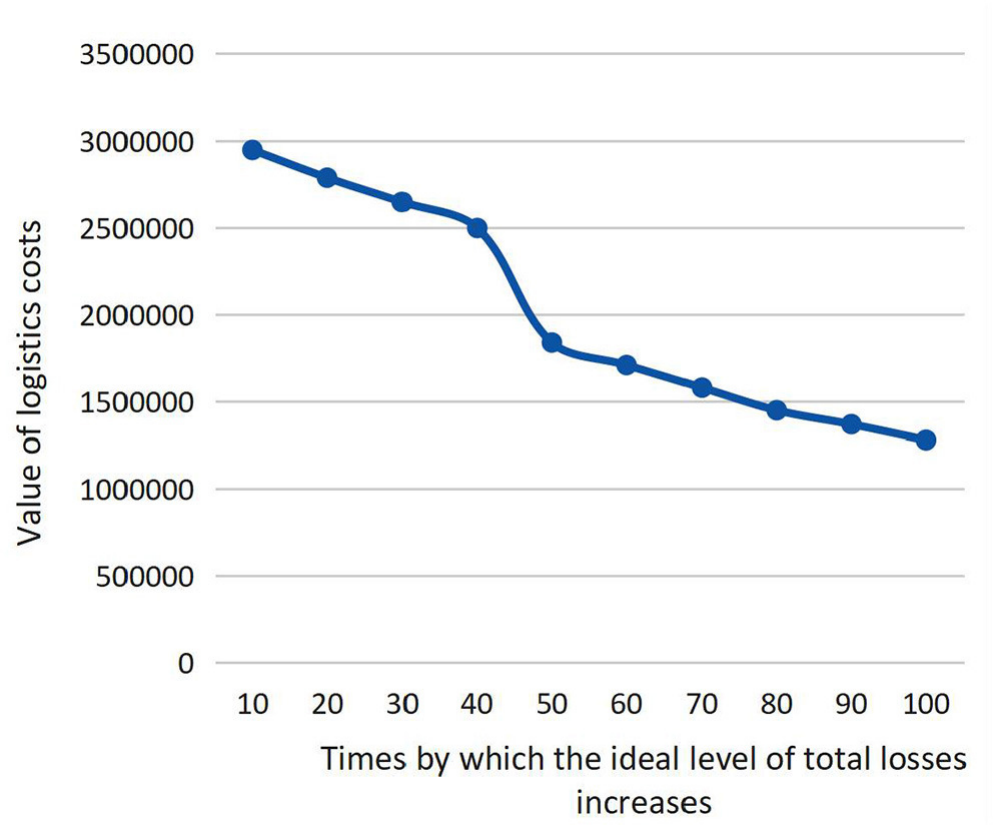
Tradeoffs between the two objectives.

To verify the rationality of the best solution of the model, we analyze the changes of minimum logistics costs (second objective function) by increasing the ideal level of total losses (first objective function), which are depicted in Figure 5. Obviously, each dot in Figure 5 represents a tradeoff between two conflicting objectives, and the fitted curve linking all the dots forms the Pareto Front. From a traditional viewpoint, it is hard to say which one is better between any two dots in the Pareto Front. However, the minimum logistics cost decreases mildly when the ideal level of total losses increases. Even the ideal level of total losses increases by 100 times (that is, the minimum total losses are RMB 12.6 billion), the minimum logistics costs only decrease by 1.67 times (RMB 1.91 million). This implies that the increase of total losses cannot significantly decrease logistics cost. In other words, saving a certain amount of logistics costs will cause a dramatic increase of victims' losses, which is not acceptable because social welfare must be superior to economic burdens in humanitarian-aid practice. Nevertheless, the tradeoff between the two objective functions are reasonable in the abovementioned numerical results.

### The Merit of the Improved B&B Method

The merit of the improved B&B method mainly lies in three aspects.

**Figure 6:**
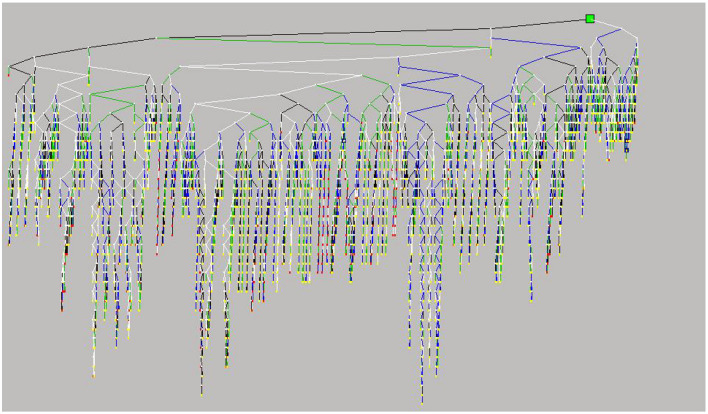
Most complicated B&B tree in this study.

First, the improved B&B method avoids the exponential growth of the B&B tree. The B&B tree is mostly not large, even if the most complicated B&B tree (which emerges when the ideal level of total losses increases by 100 times) is acceptable (see Figure 6). The reason is that many advanced technologies are employed as the core components in the Xpress-MP, such as pre-solving, cutting planes, branching variable selection, node preprocessing, and heuristics. These technologies continuously act on the formation and evolution of the B&B tree and significantly influence the solution time.

Second, the solution efficiency is given a big boost by the improved B&B method. We compare it with the traditional B&B method to respectively deal with the numerical case on the premise of the same settings (i.e. *R*_1_ = 1.26358*e*+8, *R*_2_ = 0, and λ_1_>>λ_2_). Benefiting from pre-solving and other advanced technologies, the improved B&B leads to much smaller-scale constraints, variables, and non-zero elements, and the resulting much less runtime than the traditional B&B. It is worth noting that, this significant improvement of solution efficiency is not on the premise of sacrificing the solution accuracy. The solution accuracy still stays unchanged (see Table 5).

**Table 5:** Comparisons between traditional and improved B&B.

	**Constraints**	**Variables**	**Non-zero elements**	**Best solution status**	**Best solution error**	**Runtime (s)**
Traditional B&B	385	259	1397	Global opt.	0	12
Improved B&B	109	191	805	Global opt.	0	0.1
	↘71.7%	↘26.3%	↘42.4%	→	→	↘99.2%

**Figure 7:**
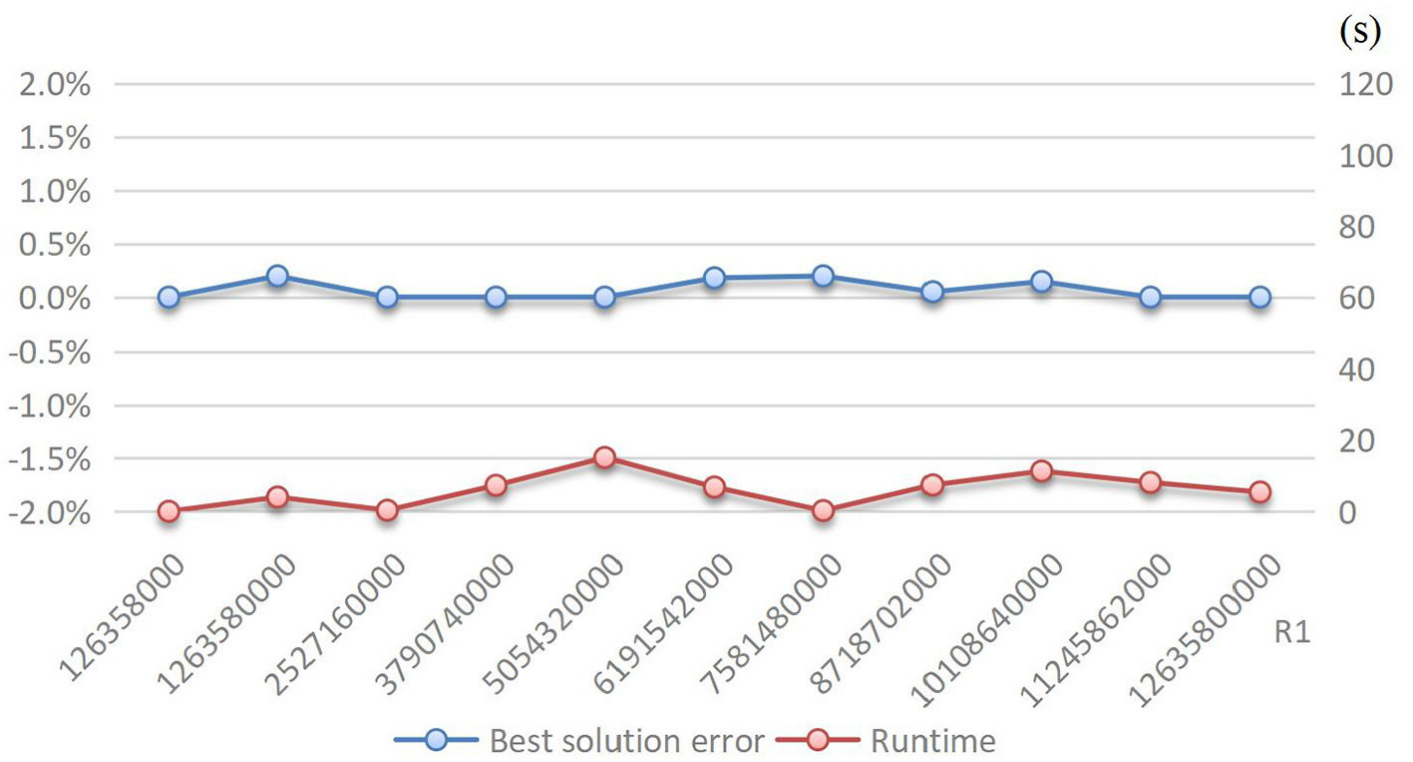
The steadiness of method under different parameter settings.

Third, the result robustness is proved good by the improved B&B method. Recall that in Figure 5, the curve of Pareto Front results from the gradual increase of the ideal level of total losses. Therefore, it is proper to regard the ideal level of total losses as a key parameter. Then, we conduct a sensitivity analysis with respect to the ideal level of total losses to show its effect on solution error and runtime. As shown in Figure 7, when the ideal level of total losses gradually increases from its original value to 100-times expansion, the best solution error and runtime are both very close to zero, and their changing curves both keep flat with slight fluctuation. More precisely, the best solution error ranges only from 0 to 0.2%, whereas the runtime ranges only from 0 to 20 s. This implies that the improved B&B method can ensure the result robustness regardless of which tradeoff reached by the total losses and logistics cost. This is very important for humanitarian logistics practice. The steady and high-quality performance of not only accuracy but also efficiency of the improved B&B method to solve the numerical case agrees with the requirements on humanitarian logistics.

## Conclusions

EID is an unforeseeable public health emergency, therefore tackling the dynamics issue and putting forward reasonable and efficient plans for humanitarian logistics is an important research topic. This study examines the decision-making problem for locating the DCs and allocating PPEs to victims based on victims' bearing capacity evolution. In a three-layer network composed of reserve centers, DCs, and affected areas, the location of DCs and allocation of PPEs are simultaneously optimized. Logistics time and demand satisfaction level are incorporated to build a loss function on the victims' side. Moreover, a multi-objective optimization model is formulated and applied to a case study. The following are the conclusions and insights.

First, the optimal level of the compromise between fairness and timeliness is reached, because the optimal result of first objective fully reaches the ideal level. The loss function plays an important role on leading to this result, which can help the decision maker put forward a reasonable, optimal humanitarian logistics plan in practice.

Second, minimizing victims' total losses is superior to minimizing logistics costs. The decrease of the ideal level of victims' total losses cannot significantly improve the best value of logistics costs. This finding conforms to the characteristics of humanitarian logistics. Minimizing victims' total losses, to some extent, representing pursuing the social welfare, must be superior to economic burdens.

Third, the combined use of the goal programming approach and the improved B&B method has significant merit which includes avoiding the exponential growth of the B&B tree, improving the solution efficiency, and ensuring the good result robustness. In other words, the proposed solution approach can simultaneously pursue efficiency and accuracy when facing a large-scale problem, which meets the requirements of quick response in practice.

However, this study still has several limitations. The real-world decision making involves other more practical factors. Further research can add the stochastic components to re-building the loss function and re-formulating the model. Considering a more complicated humanitarian logistics network and the three-dimensional transportation modes based on multiple transportation platforms are also an improved way. Nonetheless, we hope this study can provide an efficient and fair decision-making tool to deal with the humanitarian logistics planning for PPEs, and relatively make up the gaps existed in the present decision-making framework.

## Data Availability Statement

The original contributions presented in the study are included in the article/supplementary material, further inquiries can be directed to the corresponding authors.

## Author Contributions

S-lZ: conceptualization, resources, data curation, validation, methodology, formal analysis, project administration, writing—original draft, and visualization. XG: conceptualization, investigation, project administration, and writing—review and editing. YY and Y-CC: conceptualization, investigation, and writing—review and editing. All authors contributed to the article and approved the submitted version.

## Funding

This work was supported by National Natural Science Foundation of China (Grant No. 72072163) and Zhejiang Provincial Natural Science Foundation of China (Grant No. LY19G030004 and LY19G020003).

## Conflict of Interest

The authors declare that the research was conducted in the absence of any commercial or financial relationships that could be construed as a potential conflict of interest.

## Publisher's Note

All claims expressed in this article are solely those of the authors and do not necessarily represent those of their affiliated organizations, or those of the publisher, the editors and the reviewers. Any product that may be evaluated in this article, or claim that may be made by its manufacturer, is not guaranteed or endorsed by the publisher.

## References

[1] Besiou M Van Wassenhove LN. Humanitarian operations: a world of opportunity for relevant and impactful research. Manuf Serv Oper Manag. (2020) 22:135–45. 10.1287/msom.2019.0799

[2] Nikolopoulos K Punia S Schäfers A Tsinopoulos C Vasilakis C. Forecasting and planning during a pandemic: COVID-19 growth rates, supply chain disruptions, and governmental decisions. Eur J Oper Res. (2021) 290:99–115. 10.1016/j.ejor.2020.08.001 32836717 PMC7413852

[3] Mosallanezhad B Chouhan VK Paydar MM Hajiaghaei-Keshteli M. Disaster relief supply chain design for personal protection equipment during the COVID-19 pandemic. Appl Soft Comput. (2021) 112:107809. 10.1016/j.asoc.2021.107809 34421442 PMC8372451

[4] Rebmann T Citarella B Alexander S Russell B Volkman JC. Personal protective equipment use and allocation in home health during disasters. Am J Infect Control. (2011) 39:823–31. 10.1016/j.ajic.2011.01.014 21703717

[5] Labib A. Towards a new approach for managing pandemics: Hybrid resilience and bowtie modelling. Saf Sci. (2021) 139:105274. 10.1016/j.ssci.2021.105274 34720425 PMC8545685

[6] Fujihara S Tabuchi T. The impact of COVID-19 on the psychological distress of youths in Japan: a latent growth curve analysis. J Affect Disord. (2022) 305:19–27. 10.1016/j.jad.2022.02.055 35218863 PMC8865937

[7] Ivanov D Dolgui A. OR-methods for coping with the ripple effect in supply chains during COVID-19 pandemic: managerial insights and research implications. Int J Prod Econ. (2021) 232:107921. 10.1016/j.ijpe.2020.107921 32952301 PMC7491383

[8] Zhan S L Liu S Ignatius J Chen D Chan FT. Disaster relief logistics under demand-supply incongruence environment: a sequential approach. Appl Math Model. (2021) 89:592–609. 10.1016/j.apm.2020.07.002

[9] Binkley CE Kemp DS. Ethical rationing of personal protective equipment to minimize moral residue during the COVID-19 pandemic. J Am Coll Surg. (2020) 230:1111–3. 10.1016/j.jamcollsurg.2020.03.031 32278727 PMC7195046

[10] Kamyabniya A Noormohammadzadeh Z Sauré A Patrick J. A robust integrated logistics model for age-based multi-group platelets in disaster relief operations. Transp Res E: Logist Transp Rev. (2021) 152:102371. 10.1016/j.tre.2021.102371

[11] Eisenhandler O Tzur M. A segment-based formulation and a matheuristic for the humanitarian pickup and distribution problem. Transp Sci. (2019) 53:1389–408. 10.1287/trsc.2019.0916

[12] Wang Y Peng S Xu M. Emergency logistics network design based on space–time resource configuration. Knowl Based Syst. (2021) 223:107041. 10.1016/j.knosys.2021.107041

[13] Cao C Li C Yang Q Liu Y Qu TA. A novel multi-objective programming model of relief distribution for sustainable disaster supply chain in large-scale natural disasters. J Clean Prod. (2018) 174:1422–35. 10.1016/j.jclepro.2017.11.037

[14] Altay N Green III WG. OR/MS research in disaster operations management. Eur J Oper Res. (2006) 175:475–93. 10.1016/j.ejor.2005.05.016

[15] Caunhye A M Nie X Pokharel S. Optimization models in emergency logistics: A literature review. Socioecon Plann Sci. (2012) 46:4–13. 10.1016/j.seps.2011.04.004

[16] Özdamar L Demir O. A hierarchical clustering and routing procedure for large scale disaster relief logistics planning. Transp Res E: Logist Transp Rev. (2012) 48: 591–602. 10.1016/j.tre.2011.11.003

[17] Zhang X Zhang Z Zhang Y Wei D Deng Y. Route selection for emergency logistics management: a bio-inspired algorithm. Saf Sci. (2013) 54:87–91. 10.1016/j.ssci.2012.12.003

[18] Beraldi P Bruni ME A. probabilistic model applied to emergency service vehicle location. Eur J Oper Res. (2009) 196:323–31. 10.1016/j.ejor.2008.02.02727776309

[19] Zhan S L Liu N Ye Y. Coordinating efficiency and equity in disaster relief logistics via information updates. Int J Syst Sci. (2014) 45: 1607–21. 10.1080/00207721.2013.777490

[20] Rodríguez-Espíndola O Albores P Brewster C. Disaster preparedness in humanitarian logistics: a collaborative approach for resource management in floods. Eur J Oper Res. (2018) 264:978–93. 10.1016/j.ejor.2017.01.021

[21] Seraji H Tavakkoli-Moghaddam R Asian S Kaur H. An integrative location-allocation model for humanitarian logistics with distributive injustice and dissatisfaction under uncertainty. Ann Oper Res. (2021) 1:1–47. 10.1007/s10479-021-04003-5

[22] Moreno A Alem D Ferreira D. Heuristic approaches for the multiperiod location-transportation problem with reuse of vehicles in emergency logistics. Comput Oper Res. (2016) 69:79–96. 10.1016/j.cor.2015.12.002

[23] Duhamel C Santos AC Brasil D Châtelet E Birregah B. Connecting a population dynamic model with a multi-period location-allocation problem for post-disaster relief operations. Ann Oper Res. (2016) 247:693–713. 10.1007/s10479-015-2104-1

[24] Shavarani SM. Multi-level facility location-allocation problem for post-disaster humanitarian relief distribution: a case study. J Humanit Logist Supply Chain Manag. (2019) 9:70–81. 10.1108/JHLSCM-05-2018-0036

[25] Sheu JB. Dynamic relief-demand management for emergency logistics operations under large-scale disasters. Transp Res E: Logist Transp Rev. (2010) 46:1–17. 10.1016/j.tre.2009.07.005

[26] Sheu JB. Post-disaster relief–service centralized logistics distribution with survivor resilience maximization. Transp Res B: Methodol. (2014) 68:288–314. 10.1016/j.trb.2014.06.016

[27] Lu CC Ying KC Chen HJ. Real-time relief distribution in the aftermath of disasters–A rolling horizon approach. Transp Res E: Logist Transp Rev. (2016) 93:1–20. 10.1016/j.tre.2016.05.002

[28] Haghi M Ghomi SMTF Jolai F. Developing a robust multi-objective model for pre/post disaster times under uncertainty in demand and resource. J Clean Prod. (2017) 154:188–202. 10.1016/j.jclepro.2017.03.102

[29] Zhang J Liu H Yu G Ruan J Chan FT. A three-stage and multi-objective stochastic programming model to improve the sustainable rescue ability by considering secondary disasters in emergency logistics. Comput Ind Eng. (2019) 135:1145–54. 10.1016/j.cie.2019.02.003

[30] Cao C Liu Y Tang O Gao X. A fuzzy bi-level optimization model for multi-period post-disaster relief distribution in sustainable humanitarian supply chains. Int J Prod Econ. (2021) 235:108081. 10.1016/j.ijpe.2021.108081

[31] Uichanco J. A model for prepositioning emergency relief items before a typhoon with an uncertain trajectory. Manuf Serv Oper Manag. (2021) 24:2. 10.1287/msom.2021.0980

[32] Mohammadi S Darestani SA Vahdani B Alinezhad A. A robust neutrosophic fuzzy-based approach to integrate reliable facility location and routing decisions for disaster relief under fairness and aftershocks concerns. Comput Ind Eng. (2020) 148:106734. 10.1016/j.cie.2020.106734

[33] Jia H Ordonez F Dessouky MM. Solution approaches for facility location of medical supplies for large-scale emergencies. Comput Ind Eng. (2007) 52:257–76. 10.1016/j.cie.2006.12.007

[34] Mete HO Zabinsky ZB. Stochastic optimization of medical supply location and distribution in disaster management. Int J Prod Econ. (2010) 126:76–84. 10.1016/j.ijpe.2009.10.00428580891

[35] Sheu J Pan C. A method for designing centralized emergency supply network to respond to large-scale natural disasters. Transp Res B: Methodol. (2014) 67:284–305. 10.1016/j.trb.2014.05.011

[36] Zaric GS Brandeau ML. Dynamic resource allocation for epidemic control in multiple populations. Math Med Biol. (2002) 19:235–55. 10.1093/imammb/19.4.235 12828363

[37] Wang H Wang X Zeng AZ. Optimal material distribution decisions based on epidemic diffusion rule and stochastic latent period for emergency rescue. Int J Math Oper Res. (2009) 1:76–96. 10.1504/IJMOR.2009.022876

[38] He Y Liu N. Methodology of emergency medical logistics for public health emergencies. Transp Res E: Logist Transp Rev. (2015) 79:178–200. 10.1016/j.tre.2015.04.007 32288598 PMC7147567

[39] Büyüktahtakin IE. des-Bordes E, Kibis EY. A new epidemics–logistics model: insights into controlling the ebola virus disease in West Africa European. J Oper Res. (2018) 265:1046–63. 10.1016/j.ejor.2017.08.037

[40] Qin R Liao H Jiang L. An enhanced even swaps method based on prospect theory with hesitant fuzzy linguistic information and its application to the selection of emergency logistics plans under the COVID-19 pandemic outbreak. J Oper Res Soc. (2021) 4:1–13. 10.1080/01605682.2021.1897485

[41] Tzeng GH Cheng HJ Huang TD. Multi-objective optimal planning for designing relief delivery systems. Transp Res E: Logist Transp Rev. (2007) 43:673–86. 10.1016/j.tre.2006.10.012

[42] Vitoriano B Ortuño MT Tirado G Montero J. A multi-criteria optimization model for humanitarian aid distribution. J Glob Optim. (2011) 51:189–208. 10.1007/s10898-010-9603-z

[43] Huang M Smilowitz K Balcik B. Models for relief routing: Equity, efficiency and efficacy. Transp Res E: Logist Transp Rev. (2012) 48:2–18. 10.1016/j.tre.2011.05.004

[44] Tofighi S Torabi SA Mansouri SA. Humanitarian logistics network design under mixed uncertainty. Eur J Oper Res. (2016) 250:239–50. 10.1016/j.ejor.2015.08.059

[45] Li S Ma Z Teo KL. A new model for road network repair after natural disasters: Integrating logistics support scheduling with repair crew scheduling and routing activities. Comput Ind Eng. (2020) 145:106506. 10.1016/j.cie.2020.106506

[46] Arnette AN Zobel CW. A risk-based approach to improving disaster relief asset pre-positioning. Prod Oper Manag. (2019) 28:457–78. 10.1111/poms.12934

[47] Zhou Y Liu J Zhang Y Gan X. A multi-objective evolutionary algorithm for multi-period dynamic emergency resource scheduling problems. Transp Res E: Logist Transp Rev. (2017) 99: 77-95. 10.1016/j.tre.2016.12.011

[48] Sun H Wang Y Xue Y. A bi-objective robust optimization model for disaster response planning under uncertainties. Comput Ind Eng. (2021) 155:107213. 10.1016/j.cie.2021.107213

[49] Dalal J Üster H. Robust emergency relief supply planning for foreseen disasters under evacuation-side uncertainty. Transp Sci. (2021) 55:791–813. 10.1287/trsc.2020.1020

[50] Chang FS Wu JS Lee CN Shen HC. Greedy-search-based multi-objective genetic algorithm for emergency logistics scheduling. Expert Syst Appl. (2014) 41:2947–56. 10.1016/j.eswa.2013.10.026

[51] Feng JR Gai W Li J. Multi-objective optimization of rescue station selection for emergency logistics management. Saf Sci. (2019) 120:276–82. 10.1016/j.ssci.2019.07.011

[52] Wei X Qiu H Wang D Duan J Wang Y Cheng TCE. An integrated location-routing problem with post-disaster relief distribution. Comput Ind Eng. (2020) 147:106632. 10.1016/j.cie.2020.106632

[53] Li Y Zhang J Yu G. A scenario-based hybrid robust and stochastic approach for joint planning of relief logistics and casualty distribution considering secondary disasters. Transp Res E: Logist Transp Rev. (2020) 141:102029. 10.1016/j.tre.2020.102029

[54] Lawler EL Wood DE. Branch-and-bound methods: a survey. Oper Res. (1966) 14:699–719. 10.1287/opre.14.4.699

[55] Laundy R Perregaard M Tavares G Tipi H Vazacopoulos A. Solving hard mixed-integer programming problems with Xpress-MP: A MIPLIB 2003 case study. INFORMS J Comput. (2009) 21:304–13. 10.1287/ijoc.1080.0293

[56] Zhan S Liu N. Determining the optimal decision time of relief allocation in response to disaster via relief demand updates. Int J Syst Sci. (2016) 47:509–20. 10.1080/00207721.2014.891665

